# Magnetic resonance imaging using a nonuniform Bo (NuBo) field-cycling magnet

**DOI:** 10.1371/journal.pone.0287344

**Published:** 2023-06-15

**Authors:** Kartiga Selvaganesan, Yuqing Wan, Yonghyun Ha, Baosong Wu, Kasey Hancock, Gigi Galiana, R. Todd Constable

**Affiliations:** 1 Department of Biomedical Engineering, Yale University, New Haven, CT, United States of America; 2 Department of Radiology and Biomedical Imaging, Yale School of Medicine, New Haven, CT, United States of America; 3 Department of Electrical Engineering, Yale University, New Haven, CT, United States of America; King’s College London, UNITED KINGDOM

## Abstract

Magnetic resonance imaging (MRI) is a powerful noninvasive diagnostic tool with superior soft tissue contrast. However, access to MRI is limited since current systems depend on homogeneous, high field strength main magnets (B_0_-fields), with strong switchable gradients which are expensive to install and maintain. In this work we propose a new approach to MRI where imaging is performed in an inhomogeneous field using radiofrequency spatial encoding, thereby eliminating the need for uniform B_0_-fields and conventional cylindrical gradient coils. The proposed technology uses an innovative data acquisition and reconstruction approach by integrating developments in field cycling, parallel imaging and non-Fourier based algebraic reconstruction. The scanner uses field cycling to image in an inhomogeneous B_0_-field; in this way magnetization is maximized during the high field polarization phase, and B_0_ inhomogeneity effects are minimized by using a low field during image acquisition. In addition to presenting the concept, this work provides experimental verification of a long-lived spin echo signal, spatially varying resolution, as well as both simulated and experimental 2D images. Our initial design creates an open MR system that can be installed in a patient examination table for body imaging (e.g., breast or liver) or built into a wall for weighted-spine imaging. The proposed system introduces a new class of inexpensive, open, silent MRIs that could be housed in doctor’s offices much like ultrasound is today, making MRI more widely accessible.

## Introduction

Magnetic resonance imaging (MRI) is a powerful non-invasive diagnostic tool, with unparalleled tissue contrast. Over the last 30 years, the availability of MRI has significantly increased in the developed world where it has become the reference standard for diagnosis and treatment monitoring of many diseases. However, access to MR scanners is extremely limited worldwide; overall only 10% of the world’s population has access to MRI scanners [1]. Much of this discrepancy comes from the high cost associated with installing and maintaining MRI equipment. Conventional MRI systems rely on high field strengths, a homogeneous main magnetic field (B_0_-field), and strong linear switchable field gradients. The main superconducting magnet requires a dedicated room with maintenance-intensive cryogenic equipment that can incur substantial cryogen replenishment costs (particularly problematic with the current helium shortage), and expensive service contracts. Switchable gradient coils that are conventionally used for spatial encoding, also require expensive infrastructure, and dedicated chilled water sources for cooling.

Since its advent as a clinical tool much of the development work in MRI has aimed at increasing image quality or reducing scan time, and these developments have been facilitated by moving to higher B_0_-field strengths. However, the need to reduce the cost of, and increase access to MRI worldwide has renewed interest in low-field MRI.

There have been many recent industrial and academic initiatives in the development of low-field MR systems. Superconducting low-field systems currently on the market are whole body scanners that operate at 0.55T [2] or 0.5T [3, 4]. Examples include the Evry^TM^ scanner by Synaptive Medical (Toronto, Canada) which is a cryogen-free, conduction cooled system, and Paramed’s (Genoa, Italy) 0.5T open MRI system (MROpen, Paramed®) that uses a high temperature MgB2 superconducting wire to eliminate the need for helium in cryostat. By eliminating the need for cryogen cooling systems, these groups can significantly reduce the installation and maintenance costs of the scanner.

Another popular option for a cryogen-free MRI machine is a permanent magnet system. Permanent magnet arrays can be used to generate a uniform B_0_ field without any heat dissipation or electric power consumption, with the added advantage of compact size and reduced cost [5]. Many commercially available low-field MRIs are composed of permanent magnet systems, such as Aspect Imaging’s 1T scanner for use in neonatal ICUs (Embrace®, Nashville, Tennessee), Promaxo’s (Oakland, CA) 65mT office-based scanner for prostate imaging and guided intervention [6], as well as Hyperfine’s (Guilford, CT) 64mT portable brain scanner [7].

In addition to industrial initiatives, many academic efforts have also aimed at implementing permanent magnets in the form of Halbach arrays for low-field brain imaging [8–10]. While these systems have been shown to be useful in designing portable MRIs with small fringe-fields, they have limited signal due to low B_0_-fields and still require highly homogeneous main magnetic fields. Some of the first whole body clinical MRI scanners were made from permanent magnets but weight constraints limited the maximum field to approximately 0.3T.

Besides superconducting and permanent magnet arrays, low-field scanners have also been developed using resistive magnets [11, 12]. Early resistive magnet systems suffered from high power requirements and excessive heating limiting the achievable field strength [13], problems which were substantially reduced with prepolarized MRI [14–17]. This technology, first discussed for MRI applications in 1993 by Macovski and Conolly, uses a strong magnetic field to provide initial polarization prior to image acquisition at a much lower field [18]. In this way, MR signal is acquired at a lower field while still taking advantage of the SNR provided by the high polarization field. Another advantage of this system is that the uniformity of the polarizing field is not particularly important, so compact, and inexpensive magnet systems can be designed with low power requirements as is demonstrated below, in this study.

While there has been recent renewed interest in low-field MRI, most of the systems have largely focused on maintaining the traditional closed-bore geometry and using cylindrical linear gradient coils for spatial encoding which tend to increase patient discomfort especially for those with claustrophobia. Claustrophobia can be essentially eliminated with open MR systems, that discard cylindrical gradient coils for spatial encoding.

Gradient-free approaches to spatial encoding have been previously studied like Transmit Array Spatial Encoding (TRASE) [19]. TRASE imaging uses a uniform B_1_ magnitude and linearly varying radiofrequency phase to encode spatial information [20]. However, most of these applications aimed at producing a linearly varying phase with the B_1_-fields which is difficult to achieve, and the design of such a system would be much simpler if the linearity constraint is removed, since RF coils produce inherently nonlinear phase patterns [21, 22]. On the other hand, encoding methods that do take advantage of nonlinear spatial encoding fields [23–27] tend to maintain cylindrical geometry, which does not allow for an open magnet system. A technique that meets both the nonlinearity and open magnet design requirement is spatial encoding using the Bloch-Siegert shift, a phenomenon in which an off-resonance pulse induces a spatially dependent phase shift allowing for image mapping [28]. With this technique, multiple encoding coils can be used to create nonlinear encoding patterns that can be resolved using algebraic reconstruction [29].

Here we explore a new approach to MR imaging that eliminates the need for spatial encoding gradient coils and uniform magnetic fields. We present the design of a proof of concept, novel, low-field open MRI scanner consisting of a rampable nonuniform B_0_ (NuBo) main magnetic field. This field is generated by a single sided electromagnet. Unlike other single sided electromagnets that image over a small FOV because of B_0_ homogeneity requirements [30, 31], the NuBo scanner utilizes a rampable magnet that allows for prepolarization at a high field to maximize signal, and image acquisition at a lower field. In this way, the effects of the intrinsic B_0_ inhomogeneity are minimized, and the scanner maintains a cost-effective open design with a large FOV suitable for body imaging. The system takes advantage of the Bloch-Siegert shift for gradient-free nonlinear spatial encoding, and the results of this study demonstrate the feasibility of imaging in a single sided, low-field, open magnet system with an inhomogeneous B_0_-field.

## Methods

### Scanner prototype

**Fig 1:**
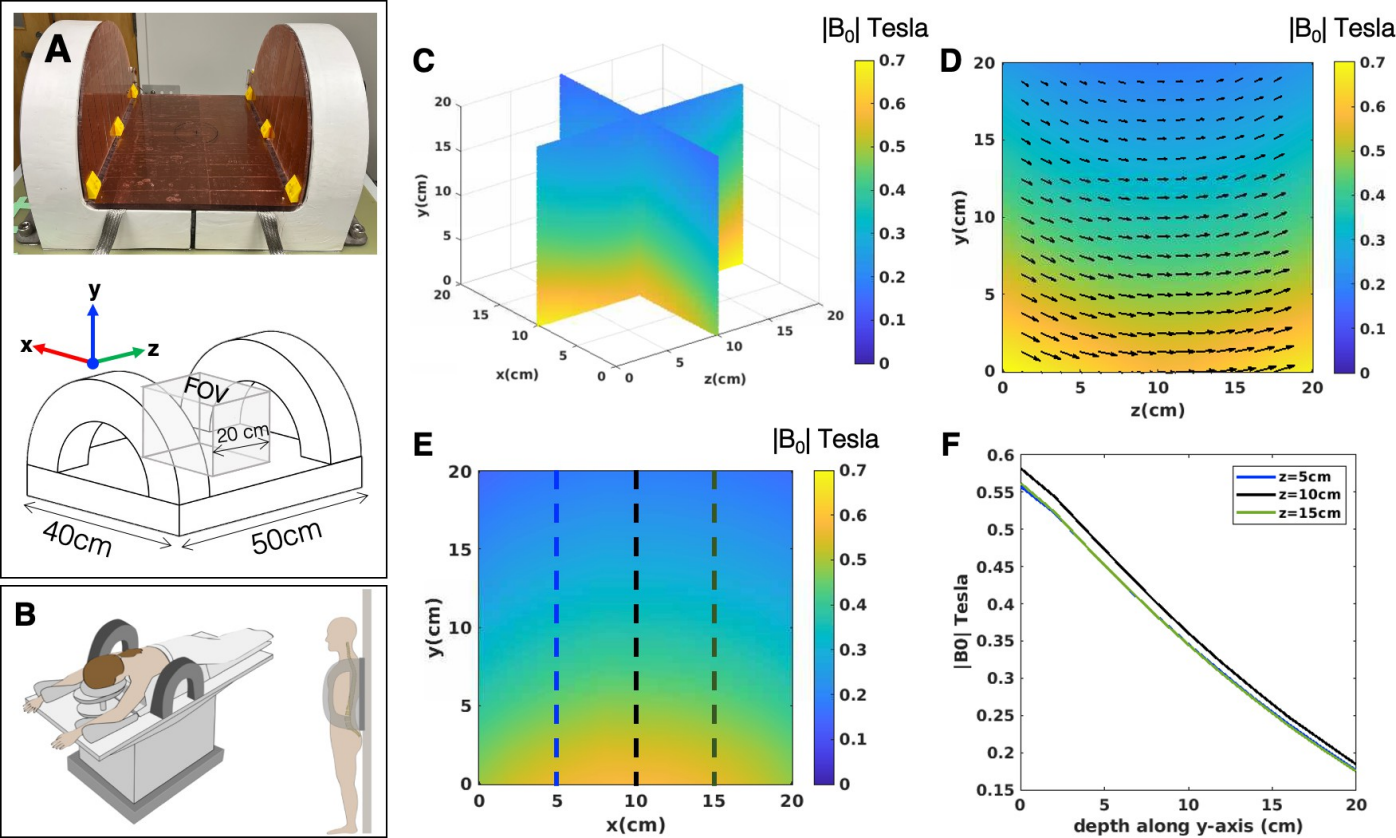
NuBo magnet design. A) Prototype of the two-element electromagnet, as well as a diagram showing the axis orientations and FOV of interest (20cmx20cm). The system is suitable for many different clinical applications including B) breast or liver MRI, as well as spinal imaging. C) 3D illustration of the magnitude B_0_-field created by the NuBo system at max power to the coil. D) The magnetic field gradient in the yz-plane overlaid with a vector field representing the direction of the magnetization. The z-component is the major contributor to the magnetic field. E) The magnetic field gradient in the xy-plane. F) Graph showing the magnetic field change with depth, along 3 different points in the FOV (dotted lines in E).

Fig 1A shows the NuBo magnet design, a single-sided imaging system featuring a non-uniform main magnetic field. The inside of the magnet is 40cm in the x-direction and 50cm in z-direction (Fig 1A). The main magnetic field is created by two sets of coil elements constructed of hollow copper wire and is pointed in the z-direction. Using hollow conductors with deionized water running through them ensures high cooling efficiency and prevents overheating. The coils can carry a maximum current of 275 Amps. The configuration of a NuBo magnet allows for an open MR system that could be built into a patient exam table and moved up and down the length of the table depending on the anatomical area to be imaged. This geometry would be particularly well suited for liver or breast imaging (Fig 1B). A similar design could also be mounted vertically on a wall for weighted spine or limb imaging (Fig 1B). Overall, the NuBo magnet design could enable MRI examinations during routine doctor office visits (much like ultrasound is used today) and provide greater accessibility to the patient during interventional procedures.

### Polarization field

The NuBo magnet produces a nonuniform, rampable B_0_-field that allows for both spin polarization and slice selection. Fig 1C shows the B_0_-field map in a 20cmx20cm FOV when maximum power is applied to the magnet to polarize the spins. In this particular configuration, the electromagnet generates a field with spin polarizations ranging from 0.6 T at the magnet surface to approximately 0.2 T at a 20cm height. The maximum target imaging height is 20cm (Fig 1F).

### Imaging field and slice selection

**Fig 2:**
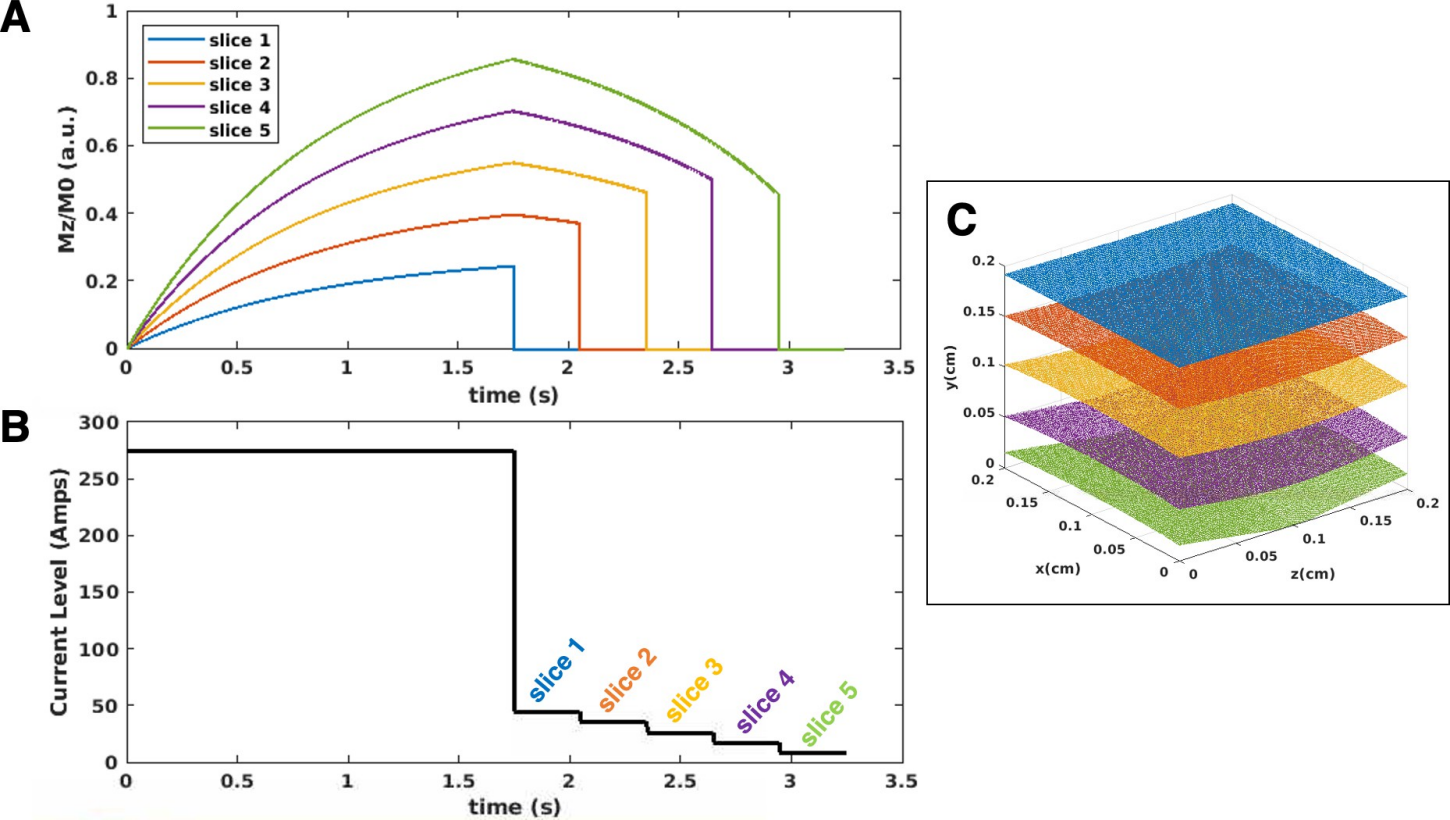
Field-cycling and prepolarization. A) Plot of the magnetization M_z_ of the curved plane over time for 5 evenly spaced slices, from a 20cm height (slice 1) to the surface of the magnet (slice 5). B) Plot showing how current to the electromagnet is adjusted in steps to achieve slice selection. C) Figure showing the location of the 5 evenly spaced slices within the FOV.

Following the spin polarization period, the rampable B_0_-field is reduced to the readout field of 24mT (1MHz) for imaging where the exact position of the imaging slice is determined by the current to the coil. In this way, the rampable B_0_-field acts much like a standard slice select gradient, except that slice selection occurs in the y-direction, and since the B_0_-field is inhomogeneous the selected imaging slice is nonplanar (Fig 2C). Slice thickness is modified by adjusting the bandwidth of the RF pulse used for excitation.

Because of the nonuniformity in the main magnetic field, the initial polarization for each slice is slightly different. Fig 2A shows how the longitudinal (M_z_) magnetization changes over time as the electromagnet is ramped between polarization and slice selection, for 5 equally spaced slices between the surface of the magnet (slice 5) and a 20cm depth (slice 1). Each line in Fig 2A represents a xz-plane within the imaging FOV (Fig 2C), with the slice closest to the surface of the magnet having the highest initial M_z_ immediately after polarization, and the slice at a 20cm height having the lowest initial M_z_. After polarization the magnet is ramped down to the readout field strength to image the first slice. When this slice is excited, encoded and acquired, M_z_ drops to zero for that slice since magnetization is flipped to the xy-plane. All other slices begin losing polarization at a rate related to the low-field tissue T1. The field is then dropped to the next slice which is excited, and the cycle is repeated until all slices are imaged. The exact excitation and acquisition parameters are flexible as in conventional MRI and will depend on the desired contrast to be generated, but the spin echo sequence simulated in this study has the following parameters: TR/TE = 300ms/16ms, 90/180 pulse duration = 60us, and Bloch-Siegert encoding pulse duration = 5.88ms, and acquisition window = 5.88ms. Fig 2B shows how current to the electromagnet is adjusted in a stepwise fashion to achieve slice selection. This field-cycling approach adds another free parameter for generating MR contrast [32–34]. For the pattern of polarization and slice ordering shown in Fig 2, the magnetization across the slice is mostly uniform despite variability in the polarization. Other slice acquisition orders and trajectories of the B_0_-ramp can be selected to balance or emphasize signal intensity, T1-weighting, or any other criteria.

Imaging in a nonuniform B_0_ is typically never done in MRI because the field inhomogeneities tend to dephase the NMR signal before it can be acquired. Great care is generally taken in the design of conventional MRI systems to ensure that the field inhomogeneity over the imaging volume varies by only a few parts per million. However, the NuBo magnet system uses a dynamic B_0_-field to polarize spins at higher field, approximately 25 times higher than the readout field of 24mT. In this scenario, the SNR is proportional to the polarizing field [20], and the homogeneity requirement of the polarizing field is significantly relaxed because dephasing of spins is negligible during polarization [18]. While the slice is nonplanar, it follows the shape of the background field, so any field inhomogeneities in the through-plane direction follow the bandwidth of the excitation pulse.

### RF encoding strategy

Spatial encoding with the NuBo system is performed with RF coils using the Bloch-Siegert shift. The Bloch-Siegert shift is a time dependent phase shift that accrues with the application of an off-resonance RF pulse. This shift is a function of the B_1_ encoding field, and is defined by the equation [35]:

$$\phi_{BS}(x,y)\;=\;\int_{0}^{\tau }\omega_{BS}(x,y)\;dt\;=\int_{0}^{\tau }\frac{|\gamma B1(x,y){|}^{2}}{2\Delta \omega_{RF}}dt\;$$ (1)

where *τ* is the duration of the RF pulse, *ω*_*BS*_ is the imposed Bloch-Siegert frequency shift, and Δ*ω*_*RF*_ is how far off-resonance the pulse is. When the B_1_-field is spatially varying, then the imposed phase shift is also spatially varying thereby creating a nonlinear encoding field. Such nonlinear RF encoding fields can be realized by applying off-resonance pulses to phased array surface coil elements, creating field patterns similar to those obtained with nonlinear B_0_-gradient encoding fields [36, 37].

Previous work has demonstrated the feasibility of using this approach to perform spatial encoding analogous to phase encoding in conventional Fourier transform based MRI [28, 35, 38]. However, most earlier applications focused on imposing linear phase shifts across the slice, which is challenging with RF encoding since the field patterns are inherently nonlinear. It is also difficult to produce sufficient encoding with RF pulses at high fields because of SAR limitations.

Therefore, embracing the nonlinear encoding patterns as well as applying the strong Bloch-Siegert pulses only during the low-field encoding and data acquisition period of the pulse sequence makes RF encoding using the Bloch-Siegert shift particularly well suited for the NuBo system.

**Fig 3:**
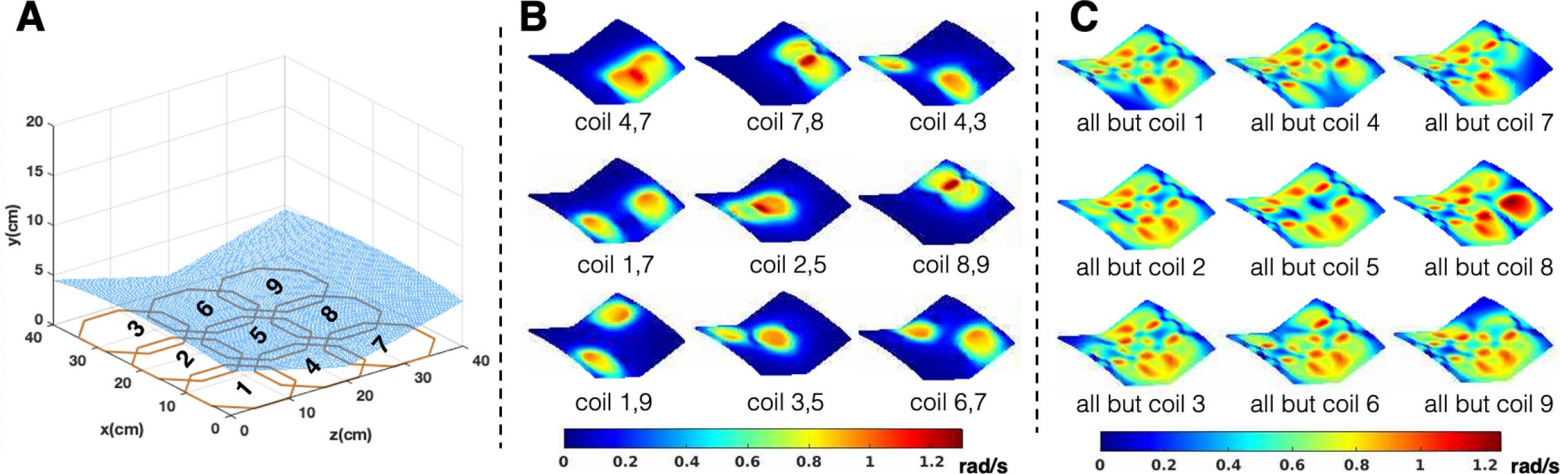
Bloch-Siegert RF encoding. A) An example of a nonplanar imaging slice selected from the inhomogeneous B_0_-field. The rampable NuBo magnet adjusts the location of the readout slices such that the entire volume can be scanned. B) and C) show a subset of 9 different nonlinear encoding patterns generated by transmitting the Bloch-Siegert off-resonance pulse on 2 or 8 coil elements, respectively. These patterns are analogous to the spatial encoding fields used in nonlinear projection imaging.

In our prototype scanner, each nonplanar slice is imaged at 1MHz (resonance frequency) and encoded using a 3x3 planar RF array. Fig 3A shows a graphical rendering of the RF array coils, and an example of a nonplanar imaging slice within the dimensions of the NuBo magnet system. RF encoding fields are created by transmitting 870kHz pulses (off-resonance frequency) on subsets of coils in the array including pairs, triplets or more coil elements, with each combination imposing unique phase patterns on the data. The relative phase of the pulse applied to each coil also affects the net encoding field, and changing the phase on different coil elements yields additional patterns. The encoding B_1_-field patterns (*B*1_*p*_) were calculated by first using the Biot-Savart Law to estimate the B_1_-field for each coil across the imaging slice, and then summing the fields for the various coil combinations:

$$B1_{p}\;=\;\sum_{1}^{\text{c}=9}\text{B}1_{\text{c}}\;*\;{\text{A}}_{\text{c}}$$ (2)

In this equation, B1_c_ is the field generated by each RF channel and A_c_ is the amplitude and phase of the Bloch-Siegert off-resonance applied to each channel.

Fig 3B and 3C show examples of different encoding patterns across the imaging slice shown in Fig 3A, from transmitting the Bloch-Siegert off-resonance pulse on 2 and 8 coils respectively. Each of these encoding patterns can be thought of as analogous to a nonlinear projection from conventional gradient fields, so the combination of many different patterns (projections) yields a nonlinear, gradient-free, encoding strategy. The elements of the array coil also serve as parallel receivers to assist in spatial localization. Using RF to perform spatial encoding in this manner eliminates the need for gradient systems, with the benefit that imaging is silent with this device.

### Image reconstruction

Image reconstruction was performed using the conjugate gradient descent method for algebraic reconstruction [39, 40]. With our RF encoding strategy the true image, ρ(*x*, *y*) is modulated by the various encoding field patterns. So, in an imaging experiment with *p* total projections, *t* sampling points, and *n* receive channels, the encoding matrix is defined as:

$$E{\left(x,y\right)}_{n,p,t}\;=\;C_{n}(x,y)\;*\;e^{i\omega_{BS}{\left(x,y\right)}_{p}*t}$$ (3)

where *C*_*n*_ (*x*, *y*) is the coil sensitivity profile for the nth receive channel, and *t* is the readout point. *ω*_*BS*_ (*x*, *y*)_*p*_ is defined in Eq 1, where the spatially varying B_1_-field for each projection is determined by Eq 2.

The corresponding signal equation for the encoding matrix in Eq 3 is:

$$s_{n,p,t}\;=\;\iint E\;{\left(x,y\right)}_{n,p,t}\;*\;\rho \;(x,y)\;dxdy$$ (4)

With the measured MR signal *s*_*n*,*p*,*t*_ and the known encoding matrix *E* (*x*, *y*)_*n*,*p*,*t*_, the true image is *ρ* (*x*, *y*) can be determined by solving the matrix inverse problem with iterative conjugate gradient minimization [41].

### Pulse sequences

**Fig 4:**
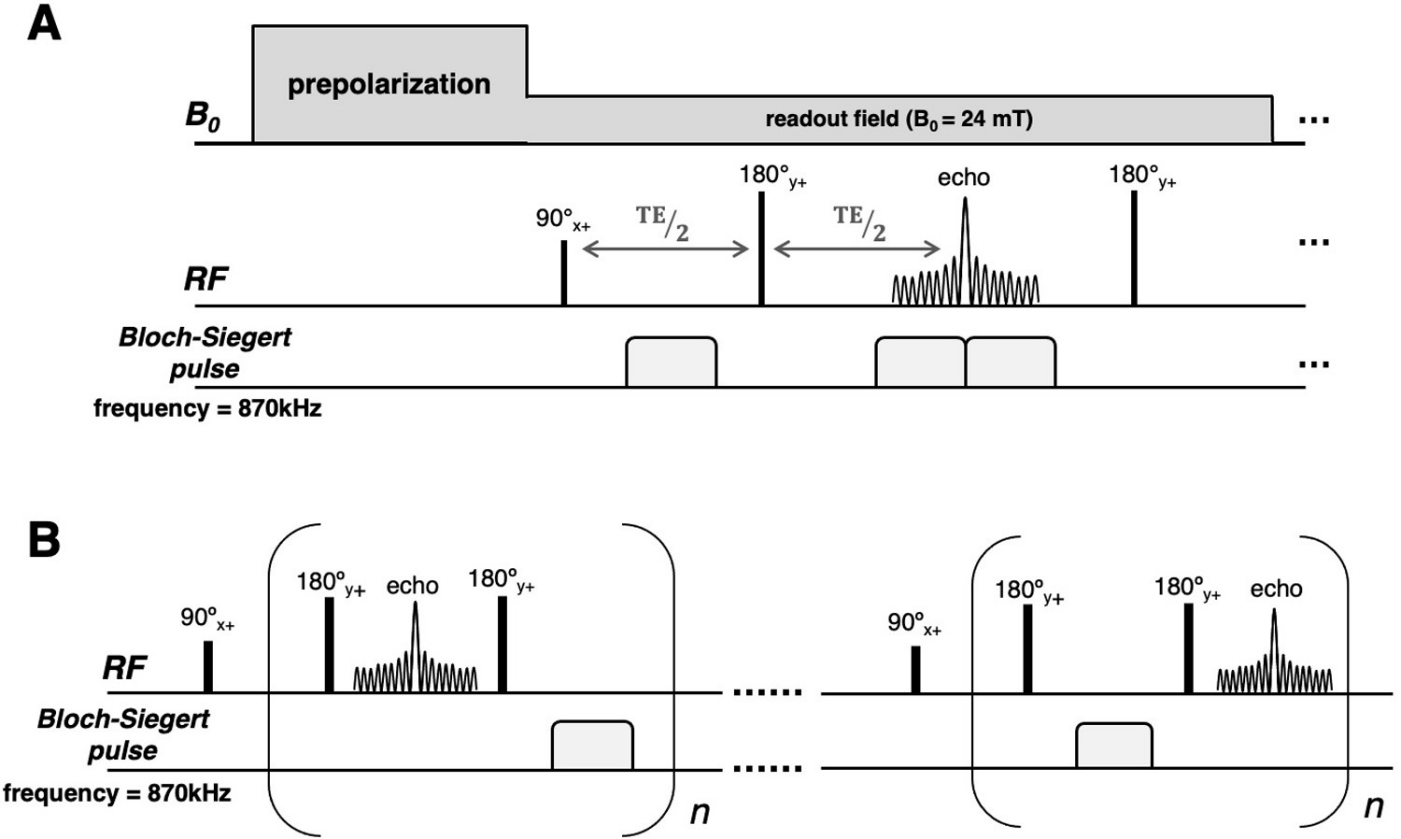
Pulse sequences. A) Spin echo sequence with Bloch-Siegert encoding pulses. The 90 and 180 pulses are applied in the low-field phase of the field-cycling, and the Bloch-Siegert pulses serve as readout gradients generating in-plane spatial encoding. B) The multi-echo encoding CPMG pulse sequence used in experiments, is designed in such a way that the Bloch-Siegert pulse acts much like a blipped phase encode gradient in turbo spin echo imaging. To collect a full k-space trajectory, the Bloch-Siegert pulses were alternatively applied to odd and even echoes.

Conventional MRI pulse sequences and contrast mechanisms are accessible for image acquisition with the NuBo system. Fig 4A shows an example of a T1-weighted encoding scheme. The sequence begins with a 90-degree excitation pulse, followed by a spin echo with a Bloch-Siegert off-resonance (Tx) pulse applied during receive (Rx), much like frequency encoding in conventional MRI. Off-resonance Bloch-Siegert encoding, and on-resonance acquisition can happen simultaneously, greatly enhancing time efficiency of the encoding. Echoes are encoded with different phase patterns imposed by the 3x3 RF array coils during successive TRs, a technique like that of nonlinear projection imaging [36, 37, 42–44]. The encoded echoes in combination with parallel receive generate sufficient data to reconstruct an image. This is the pulse sequence used in our simulation study. The spin echo sequence was simulated with

256 readout points, 256 encodings, 40cmx40cm FOV and TE = 16ms. The Bloch-Siegert pulse was applied for 5.88ms with an amplitude of 700uT.

Another way to play out the Bloch-Siegert pulse for encoding is to use a multi-echo CPMG (Carr-Purcell-Meiboom-Gill) sequence with cumulative encoding analogous to blipped echo planar imaging sequences (Fig 4B). In this sequence an excitation pulse is followed by a train of refocusing pulses interleaved with Bloch-Siegert off-resonance pulses. After acquisition of the first spin echo, each subsequent echo accumulates phase allowing for extended acquisition along a k-trajectory with each TR. To collect the positive encoding trajectory (k = 0 to k_max_), the Bloch-Siegert pulse is applied on even echoes and data is acquired on odd echoes. To collect the negative trajectory (k_-max_), the off-resonance pulse is applied to odd echoes and acquisition on even echoes. With the multi-echo encoding pulse sequence where spatial information is all phase encoded, only the echo peaks contain the relevant MR signal information. Therefore, to get symmetric k-trajectory, the echo peaks from the two TRs need to be rearranged. This sequence was used to conduct the first 2D imaging experiments. 320 echoes were acquired with an echo spacing = 1.3ms, and a total of 160 averages, noting that at this low readout field of 24mT the 180° RF pulses can be very short and of low power. The Bloch-Siegert encoding blips were applied for a duration of 350us, at maximum pulse amplitude.

### Simulation study

Bloch simulations were used to evaluate the theoretical resolution limits, and in vivo imaging capabilities of the NuBo system. Using the simulated phasors, a local k-space analysis was performed to determine the resolution limits. Unlike with linear encoding fields where the k-space coverage is uniform, with nonlinear encoding fields k-space trajectories vary based on location. With Bloch-Siegert RF encoding the resolution is dependent on the amplitude and duration of the RF pulse, so taking the spatial derivate of all phasors over the image acquisition period can be interpreted as the local k-space coverage for that region. The theoretical image spatial resolution across the imaging plane was computed using the following equations:

$$res_{x}\;=\;\underset{x}{\min}\left[\frac{1}{\frac{d}{dx}\;\left(\omega_{BS,p}(x,z)\right)}\right],\;res_{z}\;=\;\underset{z}{\min}\left[\frac{1}{\frac{d}{dz}\left(\omega_{BS,p}(x,z)\right)}\right]$$

where *ω*_*BS*_ is the Bloch-Siegert shift-based encoding pattern defined in Eq 1, and *p* is the total number of such patterns. The inverse of the first derivative in the x and z-directions of all possible encodings produces a 3D matrix of resolution maps of size [#of pixels × # of pixels × # of encoding patterns], with units of (mm/*ρ* rad). From this matrix, the minimum value at each voxel location was selected as the achievable spatial resolution at that point in the image. The phasors were also used to simulate low-field MR signal (Eq 4) and reconstruct a phantom 3T liver image. Images were reconstructed over a 256x256 matrix.

### Experimental setup

**Fig 5:**
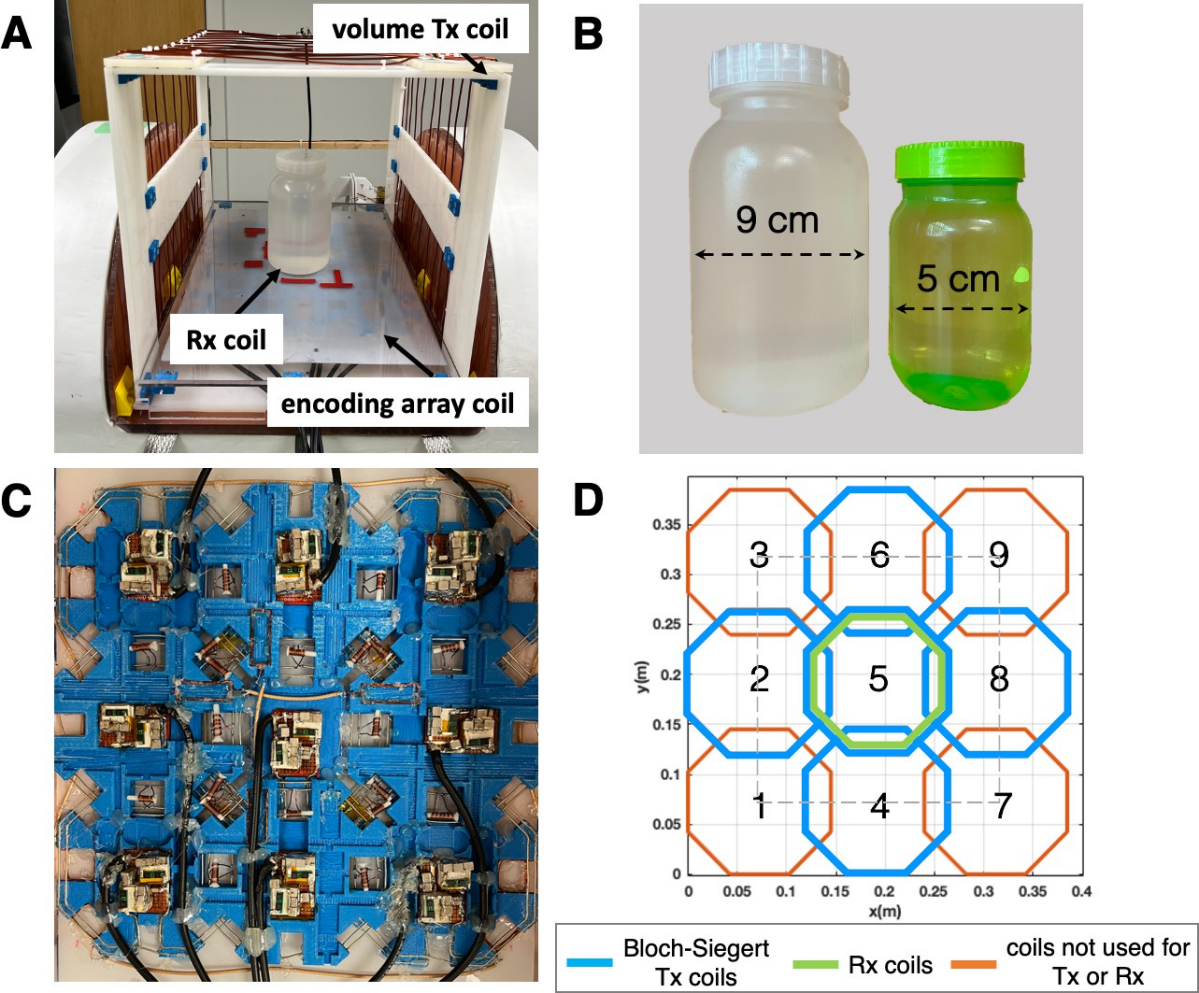
Experimental setup. A) A large Tx-only volume coil placed inside the NuBo system was used to transmit the excitation and refocusing pulses. A 3x3 RF planar array was positioned at the base of the magnet to perform spatial encoding exploiting the Bloch-Siegert shift and receive the MR signal. Data was acquired using a single channel in the center of the FOV. B) The two large water-filled phantoms with a diameter of 9cm and 5cm that were individually imaged. C) Photograph of the 3x3 RF planar array used for spatial encoding. Each element in the array is switched-tuned to an off-resonance frequency (870kHz) to transmit the high-power Bloch-Siegert pulse, and the resonance frequency (1MHz) to receive the NMR signal. D) Diagram of the array coil with the transmit channels used in experiments indicated in blue, and the receive channel in green.

Experiments were performed to demonstrate the feasibility of gradient-free imaging with the NuBo system. Fig 5A and 5B show the experimental setup and phantoms imaged, respectively. A large Tx-only volume coil placed just inside the electromagnet was used to transmit the 90 and 180 pulses; this coil generates a uniform B_1_-field within the 20cmx20cmx20cm FOV. The 3x3 RF planar array coil (Fig 5C) was positioned at the base of the magnet such that the distance between the phantom and the array were minimized, thereby optimizing encoding efficiency. The volume coil and the RF encoding array are geometrically decoupled because the former has a B_1_+-field oriented in the x-direction and the latter in the y-direction. Each element in the array was designed to passively switch between the off-resonance frequency (870kHz), and the on-resonance frequency (1MHz) [45, 46]. Two non-magnetic cross-diodes were integrated in the tuning and impedance matching circuits such that low power RF signals would drive the coils in 1MHz Rx-only mode, and high power RF pulses would switch the coils to 870kHz Tx-only mode. The return loss (S11) at both frequencies for all coil elements was below -20dB, indicating excellent tuning conditions.

Initial experiments used subsets of two and three coils from five transmit channels for nonlinear spatial encoding (Fig 5D, blue), and a single channel for receive (Fig 5D, green). Spatial encoding B_1_-field maps were estimated using the Biot-Savart law, and image reconstruction was performed with 8 encoding patterns, over a 64x64 matrix for a 20cmx20cm FOV.

## Results

**Fig 6:**
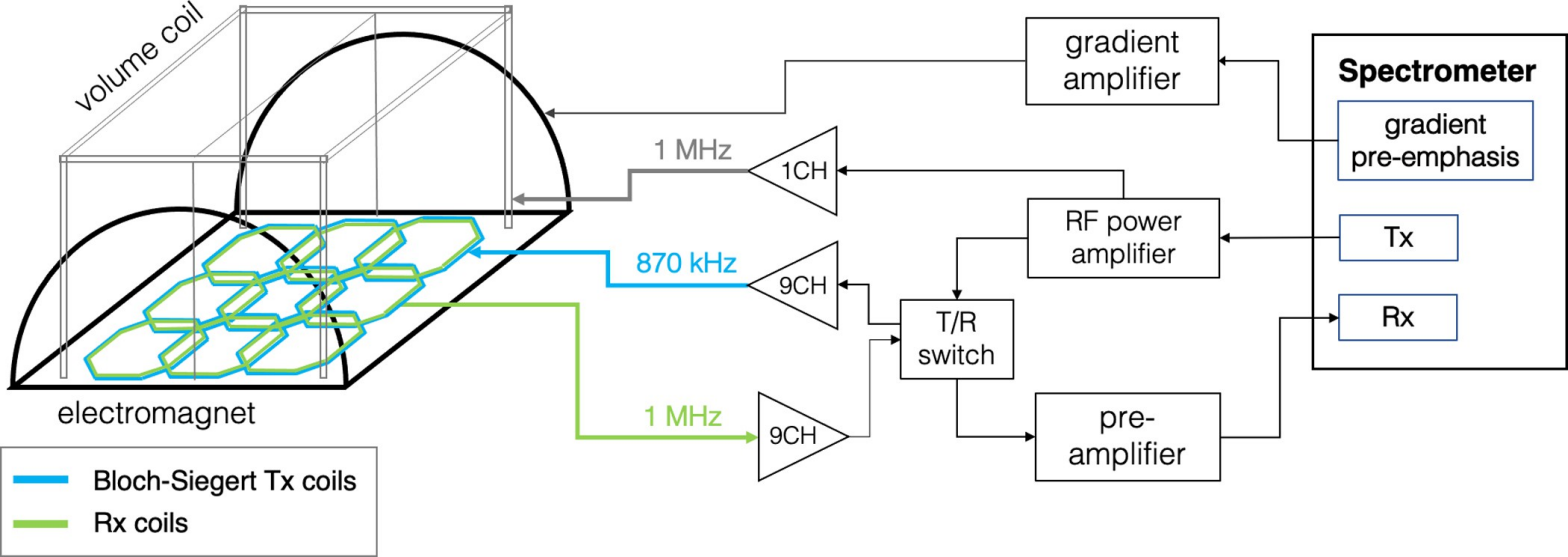
System diagram. The custom built NuBo electromagnet and supporting hardware systems. A spectrometer with 9 Tx channels and 16 Rx channels serves as the MRI console, and controls the gradient, RF, and pre-amplifiers. The necessary T/R switches for all 9 channels were built in-house. The path labeled “1CH” from the RF amplifier is for the single channel used to transmit the 1MHz excitation and refocusing pulses to the volume coil. The path labeled “9CH’’ is for the 9 channels that are used to transmit the 870kHz off-resonance Bloch-Siegert pulse and receive the 1MHz MR signal from the RF planar array.

Fig 6 illustrates the different components of the custom built NuBo magnet system (Everson Tesla, Inc, Nazareth, PA). A spectrometer (Redstone^TM^, Houston, TX) with 16 Rx channels and 9 Tx channels integrates the gradient power amplifier connected to the electromagnet (Performance Controls Inc., Montgomeryville, PA), the RF power amplifiers (Communications Power Corporation, Model AR-3-15-1E3-3C, Hauppauge, NY), and the pre-amplifiers (Ar^2^ Communications Products, Burlington, CT). The gradient amplifier (maximum output current = 275A) powers the electromagnet that is water cooled with a custom chiller from Haskris (Elmhurst, IL). The RF power amplifiers (1kW peak-power) transmit the 1MHz excitation and refocusing pulses to the large volume coil (“1CH” path in Fig 6), as well as the 870kHz high-power off-resonances pulses to the RF array coil for Bloch-Siegert encoding (“9CH” path in Fig 6). Since the array coils are designed for both RF encoding and parallel receive, a T/R switch is used to switch between the Tx and Rx paths.

**Fig 7:**
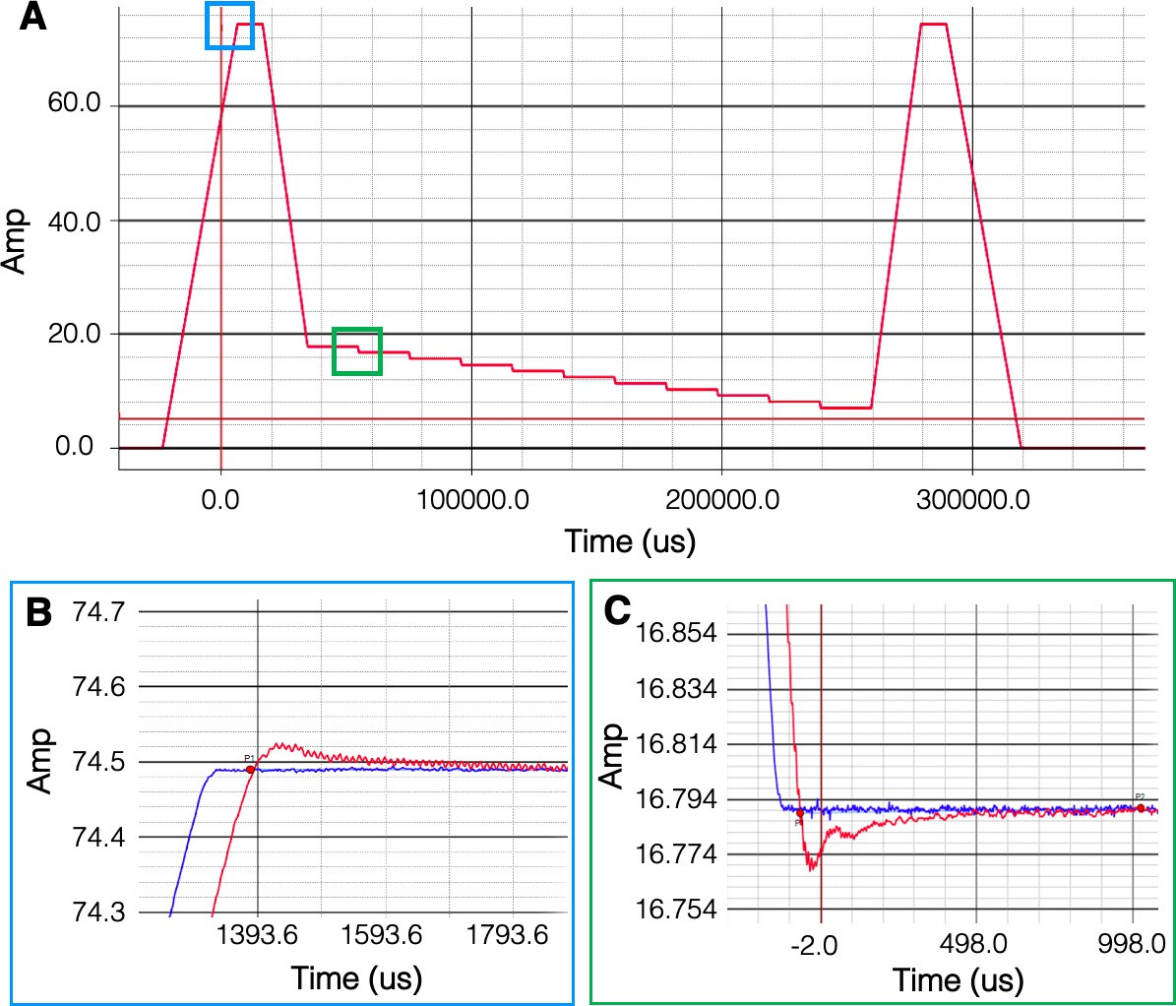
B_0_ field-cycling characterization. A) Plot showing the command signal used to drive the electromagnet (blue), and the current feedback (red). Maximum power is applied to the magnet for prepolarization, and then incremented stepwise for slice selection. B) Magnified plot at peak current (blue box), indicating a settling time of 0.8ms. C) Magnified plot at stepwise ramp down of input current (green box), indicating a settling time of 1.1ms.

As previously mentioned, the NuBo system uses an innovative field-cycling approach to maximize signal through polarization of spins at high field and spatial encoding and readout of spins at low field. Fig 7 shows the results from testing the stability of the magnet during field cycling with a maximum current of 75 Amps. Fig 7A shows the overall waveforms of the command signals (blue) and the current feedback (red); the blue plot is not visible because of the apparent alignment between the input and output waveforms. Magnification of the plot at peak current during prepolarization shows that the current takes about 0.8ms to settle (Fig 7B). The settling time for each step is approximately 1ms (Fig 7C). Both settling times, which occur while spins are along the longitudinal axis, are well within range to allow sufficient time for excitation, contrast manipulation, and spatial encoding for most pulse sequences.

**Fig 8:**
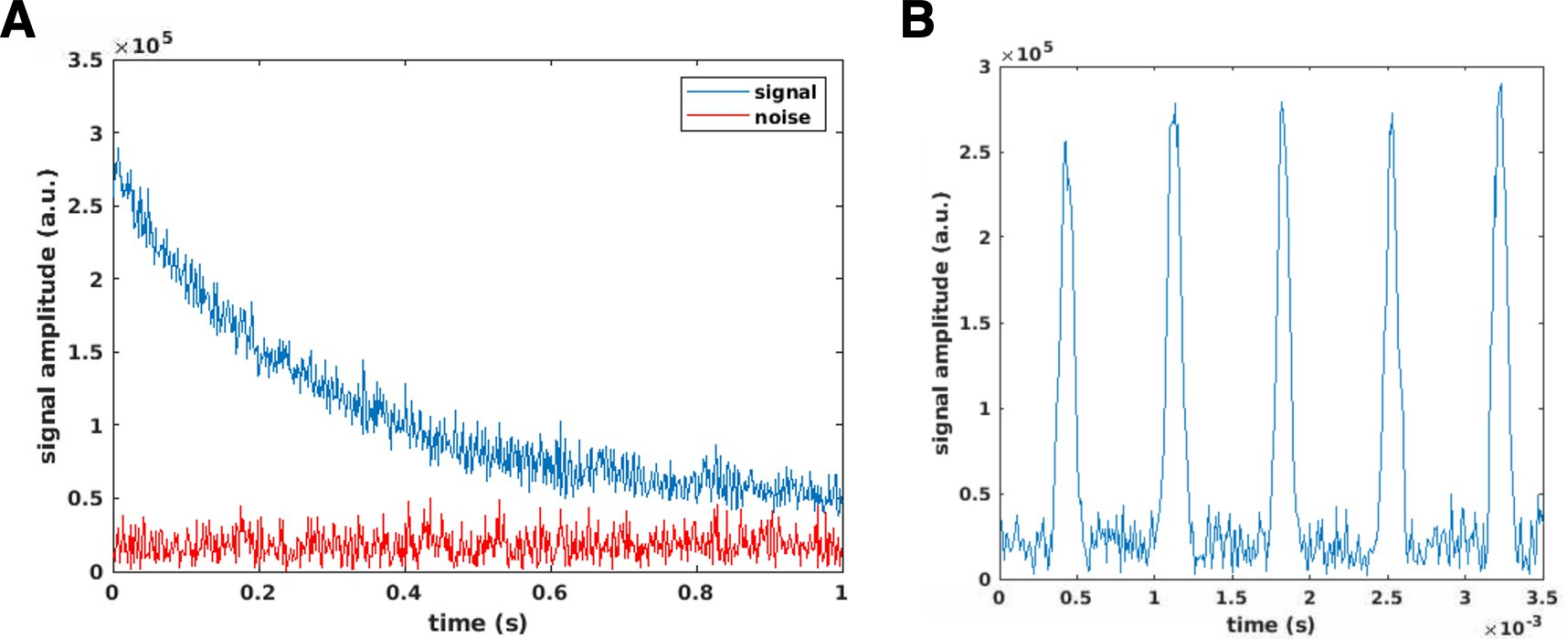
Echo train. A) Graph showing the peak amplitude for 650 echoes collected using a CPMG pulse sequence, and the experimental setup described in Fig *5*. The plot shows the signal amplitude at the echo peaks when the experiment was conducted with (blue) and without (red) a large water phantom placed inside the imaging FOV. B) Plot of the first 5 echoes in the echo train.

Fig 8 shows an echo train obtained using the experimental setup described in Fig 5. A CPMG pulse sequence with an echo spacing of 1.5ms, and no spatial encoding, was used to collect the signal train. Fig 8A shows the echo peaks for 650 echoes that were collected with (blue) and without [12] the phantom present in the imaging field-of-view, the latter represents the background noise of our experiments. Fig 8B is a plot of the first five echoes in the echo train; the acquisition window was set to 860us with a dwell time of 10us. The SNR of the echo train is about 22, calculated as the mean of the signal amplitude, divided by the standard deviation of the noise from the from the first 100 echoes in the train. As seen from these plots and the SNR calculation, the low-field readout yields long T2s with low power RF pulses; the echoes also have high signal amplitude and are well above the noise floor.

**Fig 9:**
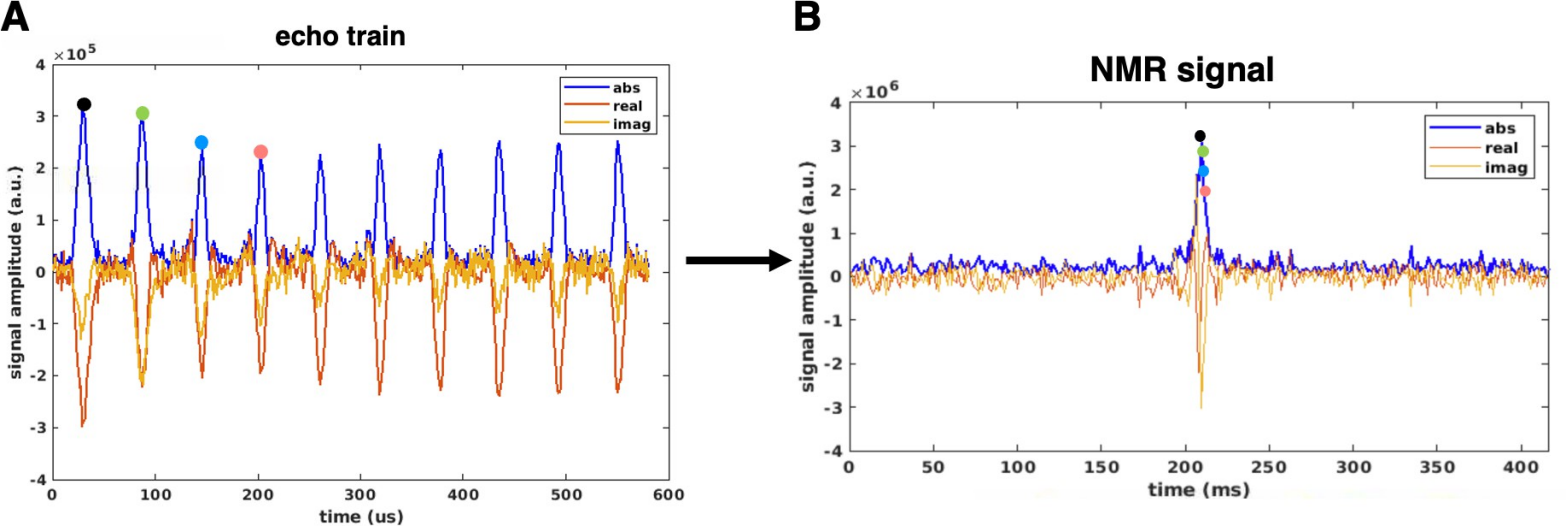
Experimental 1D MR signal. A) The first 10 echoes out of 320 echo train from a single encoding pattern. Each echo was acquired with 94 points, with a dwell time of 10us. The midpoint of each echo is used as the k-trajectory sample. B) Example of a 1D time domain MR signal formed by rearranging the midpoint of each echo.

This long echo train can be exploited for spatial encoding using the multi-echo pulse sequence previously described. Fig 9A shows an example of the first 10 experimental echoes from a positive k-trajectory echo train generated by a single encoding pattern. Each echo was acquired with 94 points, with a dwell time of 10us. The current experiments which are fully phase encoded, use only the echo peaks for spatial encoding so the time domain MR signal is formed by re-ordering the midpoints of each echo (Fig 9B). Since the spatial encoding fields are nonlinear, the echo peaks contain both k_x_ and k_y_ information.

**Fig 10:**
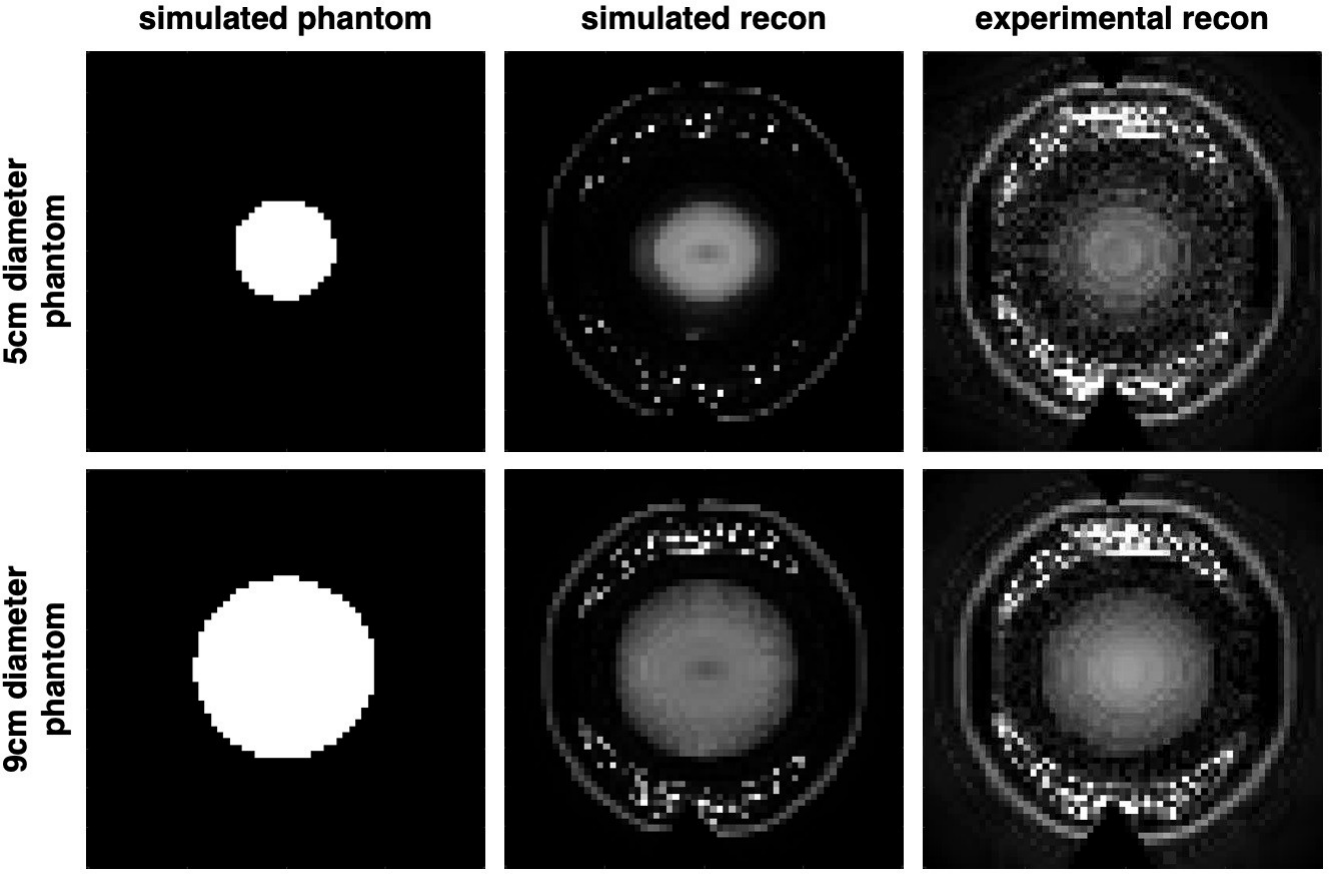
2D reconstruction. From left to right: digital phantom representing a cross section of each bottle phantom across the imaging slice, the corresponding simulated, and experimental reconstruction results.

2D experimental imaging results from reconstructing the MR signals, are shown in Fig 10. The left-most column shows the digital phantoms mimicking the cross sections of the two water-filled bottle phantoms that were imaged. For comparison, a Bloch simulation of the experiment was conducted with the same 8 encoding field patterns used in experiments. Algebraic reconstruction of the simulated and experimental signals generates the 2D images shown in the middle and right-most column of Fig 10, respectively. Images were reconstructed over a 64x64 matrix with a 20cmx20cm FOV. Image SNR calculated as the mean of the signal in the center of the bottle divided by the standard deviation of the background signal in the dark region yielded SNR = 26.94 for the 5cm phantom, and SNR = 32.81 for the 9cm phantom. These values are comparable to that of other low-field systems with field-strengths in the 20-50mT range [11, 47].

At present, the experimental data is acquired with phase encoding only and 160 averages, which extends scan time to a few hours. However, it is feasible to apply RF encoding off-resonance while simultaneously acquiring signal on resonance [48], which would significantly cut down scan time. With the appropriate hardware, we would be able to acquire at least four encodings in one TR which would reduce scan time by half for the current number of projections, and fewer averages would yield a scan time of a few minutes.

**Fig 11:**
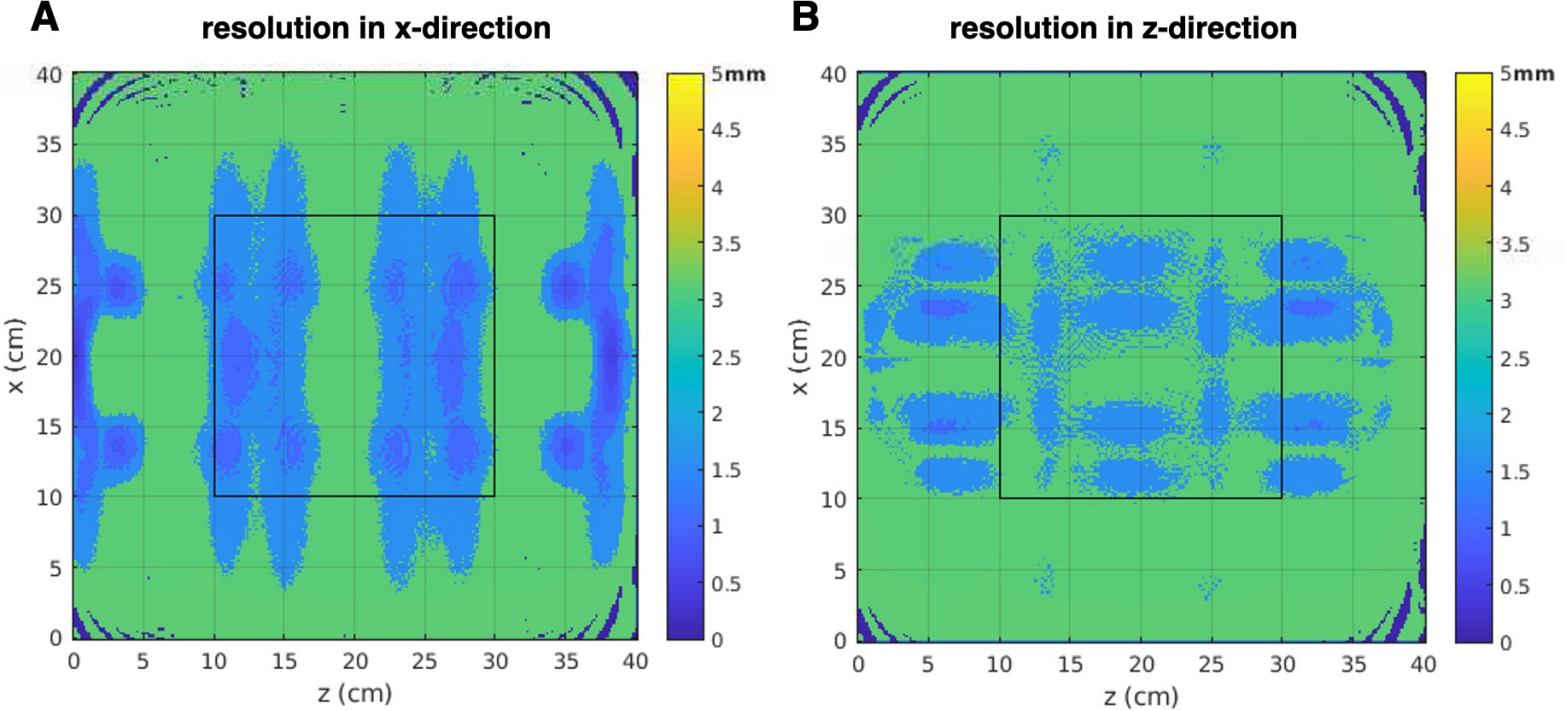
Imaging resolution analysis. The resolution achievable in the A) x-direction and B) z-direction with the encoding patterns generated by a planar 3x3 RF array coil, from applying the Bloch-Siegert pulse for 5.88ms. The black box indicates a 20cmx20cm region in the center of the FOV.

While these preliminary imaging results show that the NuBo scanner can delineate phantom geometries and boundaries, the actual resolution limits of the system were evaluated using a local k-space analysis. Fig 11 shows the achievable resolution in the x and z encoding directions at an imaging depth of 1.4cm, when the Bloch-Siegert pulse is applied for 5.88ms. On average, the minimum resolution in both directions is about 3mm.

**Fig 12:**
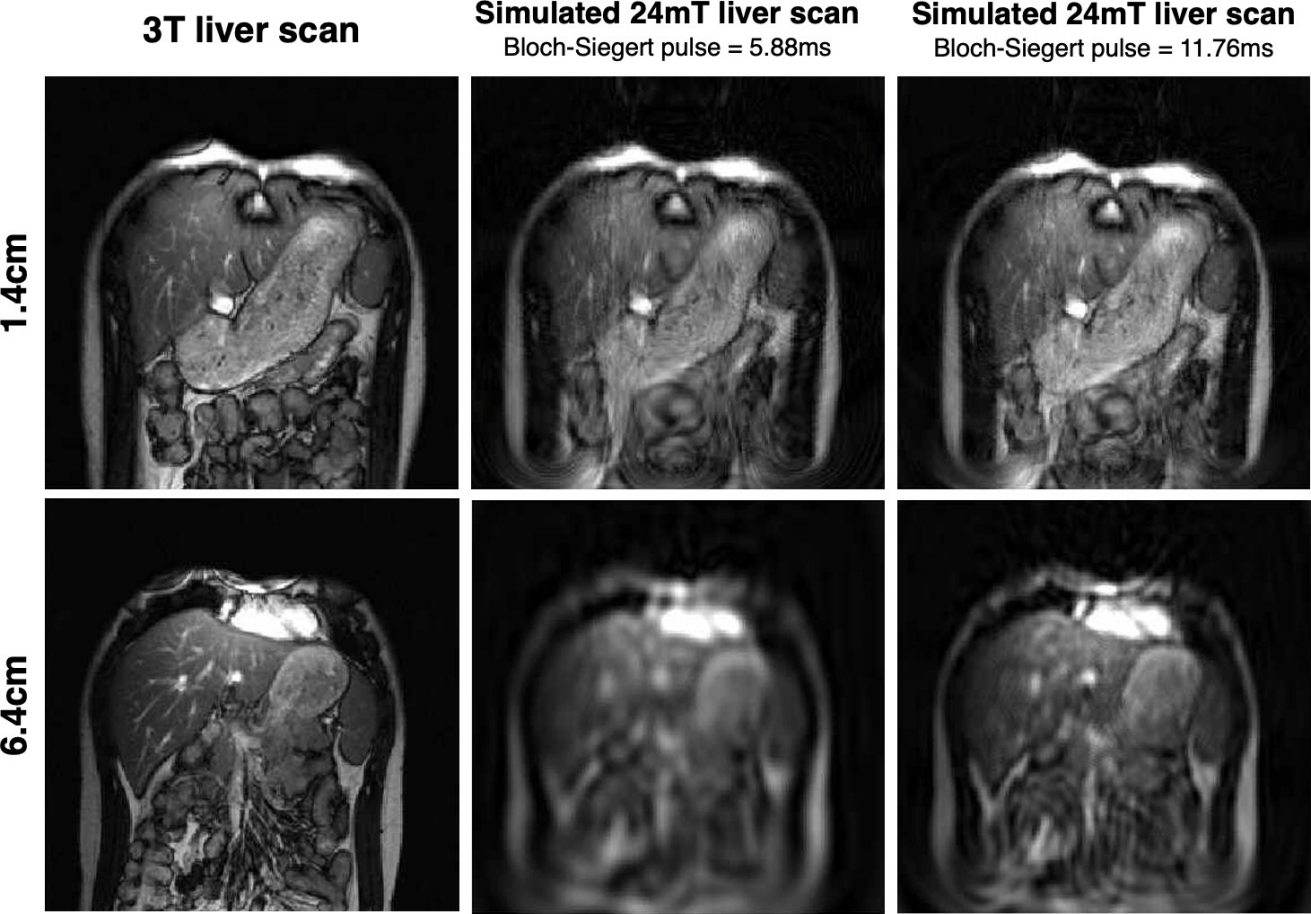
Improving SNR with depth. Simulation results demonstrating that image resolution with depth can be improved by increasing the duration of the Bloch-Siegert pulse. First column shows the high-resolution liver scans that were used as phantom images in the simulation experiments, at a 1.4cm depth (top row), and 6.4cm depth (bottom row). Middle column shows the results when the images were encoded using a Bloch-Siegert pulse with a 5.88ms length, and the last column shows the results when this pulse length was doubled to 11.76ms.

Fig 12 shows results from a simulation study evaluating Bloch-Siegert encoding capabilities of a 3x3 planar RF array in the NuBo system. High resolution 3T liver scans were reconstructed on nonplanar slices covering a 40cmx40cm FOV, using 256 encodings. The Bloch-Siegert pulse was applied for 5.88ms. Image results are shown for imaging depths of 1.4cm and 6.4cm. The depth was defined as the lowest point from the surface of the magnet, on each nonplanar slice. The SNR of the image decreases with depth (Fig 12, middle column) because the B_0_ polarization strength and the encoding B_1_-field strength drops with depth. However, the SNR of the image can be recovered by increasing the duration of the of the Bloch-Siegert pulse. Fig 12 right-most column shows simulated images that are obtained when the length of the Bloch-Siegert pulse is doubled from 5.88ms to 11.76ms. As expected, the resolution increases when the Bloch-Siegert pulse is applied for an extended period (moving further out in local k-space). The SNR is also variable across the FOV, as are contrast and resolution, but these variations are small and generally not obvious. This characteristic is similar to that of other imaging methods that incorporate nonlinear spatial encoding such as those that have been done with high field conventional magnets [36, 37, 39, 40, 42, 43, 49, 50].

## Discussion

The presented simulation studies and preliminary experiments are evidence of the feasibility of gradient-free imaging in a nonuniform B_0_-field using the novel low-field, NuBo magnet system. Two key features of this device are the field-cycling magnet, that gets around the problem of imaging with a highly nonuniform B_0_, and spatial encoding via RF, that allows us to eliminate conventional spatial encoding magnetic field gradients.

Low-field MRI is becoming an increasingly popular area of study because such systems can be built and installed with considerably lower costs than traditional high field MR systems, potentially making them more accessible. While several low-field devices are under development, most of the devices to date tend to maintain the cylindrical geometry and uniform field requirements of conventional MRI [3, 8].

By removing the constraint for a uniform B_0_-field across the imaging volume, we can design an open MRI system of almost any shape and size catered for specific imaging applications. A family of such systems could be built with specific geometries for imaging applications like breast screening, fatty liver assessment, gynecologic imaging, or weighted spine imaging. Currently, when a magnet is purchased, a range of RF coils are swapped in and out according to the imaging application. With a family of small nonuniform field systems designed for specific applications, one could envision single console and electronics, with a series of magnets and associated RF hardware selected according to the clinical need.

The NuBo system combines several pre-existing technologies previously applied in more conventional magnet systems to make imaging in a non-uniform, low magnetic field possible. These technologies include: field-cycling [51], nonlinear gradient imaging [37, 42, 43, 49, 52], RF spatial encoding [23, 53], and parallel imaging with algebraic reconstruction [54, 55]. Field-cycling makes imaging possible in a nonuniform B_0_-field by providing a high field for spin polarization (where the field inhomogeneity leads to slight differences in polarization but no dephasing), and low field for readout (where the inhomogeneity is limited to the bandwidth of the slice and decreases with readout field strength). This ensures there is minimum signal loss due to the inherent inhomogeneous B_0_-field. Moreover, SNR in field-cycling systems is proportional to the strength of the prepolarization field [20], so we are able to retain the benefits of imaging at a lower field, while harnessing the signal strength of high-field polarization. As expected, the magnetic field takes some time to stabilize when ramped up to maximum field strength for polarization and ramped down to the readout field for slice selection. However, these transients are short enough (within 1ms) that they can be easily accounted for with appropriate delays in the pulse sequence or with modified input response profiles (pre-emphasis pulses) aimed at compensating for these transients. In the work presented here, the delay between ramp down and the first excitation pulse was generously set to 100ms to avoid the effects of these transients.

The rate of polarization is governed by T1, and thus maximum polarization is achieved in approximately 5 T1’s. It is possible to manipulate polarization time as a source of contrast much in the same manner as inversion pulses in conventional MRI. Field cycling not only helps reduce the impact of nonuniformities across a slice, but also adds an additional degree of freedom to the pulse sequence that is not available with superconducting magnets. For example, specific T1’s can be nulled by reversing the direction of the B_0_ gradient during polarization, similar to inversion recovery with conventional MRI.

The low field used for readout also has advantages. Since T2 is long at low-field and very little power is required for spin refocusing, it is possible to perform multi-echo spectral imaging with many echoes to quantify T2 and diffusion effects. As shown in the Results, it is feasible to collect over 650 echoes within 1 second following a single excitation pulse. This sequence in combination with a variable first echo spacing approach [56] will allow for quantification of both diffusion and T2 [57]. In the future we will adapt the sequence for imaging such that each echo contains both spatial encoding and quantitative T2 data.

This innovative approach to MRI incorporating the use of a nonuniform rampable main magnet, greatly reduces the manufacturing specifications and tolerances for the magnet since there is no requirement of a highly uniform field–although it is important to perform precise field mapping so that the field is known. Additional hardware savings are found through the use of RF for spatial encoding allowing elimination of gradient coils and associated gradient amplifiers altogether. Studies have shown the feasibility of using RF for spatial encoding with technologies like Transmit Array Spatial Encoding (TRASE) [19] or rotating field MRI [26]. The TRASE coil produces a linearly varying B_1_-phase with a uniform magnitude. However, it is only able to produce a linear phase in a small region within the FOV; moreover, the coil design can be made much simpler without the need to achieve highly linear fields. Rotating field MRI [26], takes advantage of the nonlinearity of the B_0_-field for spatial encoding but conforms to the conventional cylindrical design. That said, it is possible and in certain situations potentially beneficial to combine this style of RF spatial encoding with spatial B_0_s-field gradients. Flat gradient coils could be added to the system but this would require additional amplifiers and take up space between the magnet and the subject reducing the field penetration depth in the subject [58]. Therefore, the Bloch-Siegert shift represents an ideal RF encoding technique for gradient-free imaging with the open magnet design of the NuBo system. Our preliminary experimental 2D imaging results demonstrate the feasibility of using the Bloch-Siegert shift effect for RF spatial encoding. The images show that we can clearly delineate phantom geometries and boundaries with just 8 encoding patterns, and image in an unshielded system. In both simulations and experiments areas of high signal intensities are seen outside the phantom region. This artifact is a result of non-bijective encoding field patterns, which means the frequency isocontours in those regions are similar to the ones in the phantom area. The quality of the reconstruction can be improved by adding more encodings and receive channels. The experimental reconstructions also contain some overall noise artifacts that most likely come from electromagnetic interferences (EMI) [59, 60]. Future work will focus on incorporating EMI correction methods [60] into signal postprocessing, as well as expanding the encoding technique to include more patterns and receive channels, for imaging over a larger FOV. To show proof-of-concept we used a large uniform volume coil to transmit the excitation and refocusing pulses as part of the experimental setup. However, future iterations of the system will use the planar RF spatial encoding array to also transmit 90/180 pulses in order to maintain the open system design.

By eliminating any linear constraints to the encoding field, we are able to create a large set of encoding patterns by simply varying the number of transmit coils and the phase of the transmit signal. Theoretical calculations have shown that the encoding capabilities of our array coil improves with a higher number of Bloch-Siegert transmit channels. To ensure we have complete flexibility in selecting transmit coils elements while maintaining access to all receive channels for maximum signal, each coil element will be designed to perform simultaneous transmit and receive.

While there are many approaches to solving this RF problem, we discuss two designs here: nested loop coils, and double-tuned coils. A nested loop coil will have a receive coil tuned to the Larmor frequency nested within a transmit coil tuned to the Bloch-Siegert off-resonance frequency. Preamplifier decoupling and RF traps can be used to reduce signal coupling between transmit and receive paths [61]. A double-tuned coil, as the name suggests, is a coil that is tuned to be resonant at both the Larmor frequency and the Bloch-Siegert off-resonance frequency so that the coil can simultaneously operate in a Tx and Rx mode [62]. To realize such a system, we have built a frequency-division duplex (FDD) switch that would allow for simultaneous high-power transmit at the off-resonance frequency and receive at the resonance frequency without any interference between the Tx and Rx channels. Either of these techniques would ultimately allow us to use the Bloch-Siegert pulse in a manner analogous to a readout gradient.

The results in Fig 12 show the imaging capabilities of the NuBo scanner with Bloch-Siegert encoding. SNR and high resolution can be obtained, and the nonplanar nature of the slices is not readily apparent. Also, the curvature of the slice is a known feature from measured B_0_-field maps, so planar slices can be extrapolated from volumetric images as a post-processing step. As expected, the overall image resolution decreases with depth because of reduced Rx sensitivity and transmit B_1_-field penetration with depth. To improve resolution for slices further away from the base of the magnet, more encoding patterns can be collected, or an additional Rx coil array can be placed on top of the patient. It should be noted that even with the nonlinear encoding patterns, the maximum resolution achievable by the NuBo system is quite uniform over the FOV (3mmx3mm), with only subtle variations in the x and z-directions. In can been seen from the simulation study that the variability in resolution in the reconstructed images is not noticeable, especially at the center in a 20cmx20cm region of interest; the soft tissue contrast is sufficient to distinguish anatomical structures. This imaging resolution is comparable or better than that of in-vivo images from other low-field scanners with potential clinical and preclinical applications [16, 30, 63].

While the spatial resolution and sensitivity of the NuBo system is less than that of a conventional high-field MRI, it will still serve as a much more accessible screening system or simple treatment monitoring device for liver or breast imaging. MR mammography is known to be superior to x-ray in terms of sensitivity, but the screening is limited to women who are known to be high risk because of cost and accessibility. There has been recent interest in developing abbreviated protocols for women at lower-risk [64], wherein this device could provide a highly accessible and inexpensive approach to mammographic screening.

Another clinical application for the NuBo system would be for non-alcoholic fatty liver disease. It has been estimated that a quarter of the world’s population suffers from non-alcoholic fatty liver disease or non-alcoholic steatohepatitis, and it appears to be an increasing problem in children and adolescents [65–67]. MRI has been found to yield quantitative fat and iron metrics that are ideal for characterizing fatty liver. This device could therefore readily produce actionable metrics at a low cost, significantly impacting the rate of diagnosis of a world health epidemic.

The NuBo scanner is not intended to replace high-field MRI systems but designed to provide easy and early access to diagnostic imaging much like that of ultrasound. Ultrasound is commonly used in doctor’s offices, it has sensitivity to a depth of 20cm or less, variable spatial resolution, much like the NuBo system, but with lower tissue contrast. With the approach proposed here, a class of MR based NuBo systems could be developed that outperform ultrasound across a wide range of applications, but at similar costs. Overall, the proposed MRI system will lead to a family of devices covering a wide range of geometries with numerous design possibilities and clinical applications.

## Conclusion

In this study we demonstrated proof-of-concept through simulations and preliminary scanner experiments that gradient-free imaging is feasible in a single-sided, nonuniform, open magnet system. The NuBo low-field magnet system re-imagines conventional MRI by moving away from the uniform B_0_-field constraint and need for gradient-based spatial encoding which will help increase access to low-cost, application specific, diagnostic MR imaging with superior soft tissue contrast. This technology will allow MRI devices to be designed in any shape or size, thereby expanding access to noninvasive diagnostic imaging to urban and rural areas across the developing world and leading to new applications. The techniques presented could significantly impact domains spanning biomedical engineering, and clinical practice, ultimately opening the door to new areas of research and innovation in the field of bioimaging.

## Supporting information

S1 File (ZIP)
