# Supplementary material for: Magnetic resonance imaging using a nonuniform Bo (NuBo) field-cycling magnet
Source: PLoS One. 2023 Jun 15;18(6):e0287344. doi: 10.1371/journal.pone.0287344 (PMC10270621; doi:10.1371/journal.pone.0287344)
Supplement: S1 File — (ZIP) [file pone.0287344.s001.zip › Simulation and Recon Scripts/Documentation.docx]

**Documentation**

This folder contains scripts to perform the Bloch simulation and conjugate gradient based image reconstructions. It also contains sample data that was used to generate the results shown in the manuscripts. The two MATLAB scripts detailed below contain the main simulation and reconstruction algorithms that can be called by the user. The rest of the files in the folder are supporting functions to these two scripts.

**generate_NMR_sig.m**

DESCRIPTION: function that uses Bloch simulations to generate the expected NMR signal from the NuBo system, based on the phantom being imaged and the applied encoding field.

INPUTS:

1) “B0_path”: string containing the path and file name of the slice profile

2) “B1Y_path”: string containing the path and file name of the y-component of the B1-field across the slice for each array coil element. The field is determined estimated from the Biot Savart law.

3) “B1X_path”: string containing the path and file name of the x-component of the B1-field across the slice for each array coil element.

4) “PE_path”: string containing the path and file name of the encoding scheme. This file contains of matrix of 0s, 1s, and -1s of size [# of encodings x 9]. 0 means that coil is not transmitting the Bloch-Siegert pulse, 1 means the coil is transmitting with phase 0, and -1 means the coil is transmitting with phase $\pi$.

5) “phantom_path”: string containing the path and file name of the phantom image

6) “output_dir”: string containing the path to save the output files

OUTPUTS:

4 files that include the simulated MR signal and encoding matrix. These files are used as inputs to the conjugate gradient reconstruction code.

EXAMPLE:

generate_NMR_sig('./slice_B0_B1_files/slice_B0_vector_1.4cm.mat','./slice_B0_B1_files/slice_B1Y_vector_1.4cm.mat','./slice_B0_B1_files/slice_B1X_vector_1.4cm.mat','./phi.mat','./liver_slice_1.4cm','./');

**Recon_Mat_File.m**

DESCRIPTION: Script that performs algebraic reconstruction of simulate or measured MR signal.

INPUTS:

1. total_Rx_sig.mat: matrix containing simulated or measured time domain MR signal for each encoding pattern and receive channel
2. wBS_shift.mat: encoding matrix of size [# if pixels x # of pixels x # of encoding patterns]
3. Rx_B1_sensitivity.mat: matrix contains the sensitivity profiles for each receiver channel
4. loop_tau_values.mat: array containing the readout time points
5. k_mat.mat: readout points in matrix form

Code to generate each of these files in included in “generate_NMR_sig.m”.

OUTPUT:

Reconstructed image
